# Clinical impact of medication adherence on 10‐year cardio‐cerebrovascular mortality in newly diagnosed hypertensive patients

**DOI:** 10.1111/jch.14320

**Published:** 2021-08-12

**Authors:** Cho‐Long Kim, Yoon‐Sung Do, Byung‐Jun Kim, Kyeong‐Soo Lee, Min‐Ah Nah, Ung Kim, Jung‐Hee Lee, Tae‐Yoon Hwang

**Affiliations:** ^1^ Yeungnam University College of Medicine Daegu Republic of Korea; ^2^ Division of Cardiology, Department of Internal Medicine Yeungnam University Medical Center, Yeungnam University College of Medicine Daegu Republic of Korea; ^3^ Department of Preventive Medicine and Public Health Yeungnam University College of Medicine Daegu Republic of Korea

**Keywords:** cardio‐cerebrovascular disease, hypertension, medication adherence

## Abstract

The purpose of this study is to evaluate the impact of medication adherence on cardio‐cerebrovascular (CCV) mortality in newly diagnosed hypertensive patients. The authors retrospectively reviewed data from 20,836 patients who newly diagnosed hypertension from January 1, 2003 to December 31, 2005. Medication adherence was calculated from the compliance ratio (CR) during the first year after the diagnosis of hypertension. CCV mortality for 10 years was assessed according to the presence or absence of complications of hypertension. The risk of CCV death was significantly reduced in the CR ≥ 70% group than in the CR < 70% group (hazard ratio, 0.70; *p* = .004) for 10 years. In the patients without complications, the risk of CCV death was significantly lower in the CR ≥ 70% group than in the CR < 70% group (hazard ratio, 0.56; *p* = .014). However, in patients with complications, there was no significant difference in risk of CCV death between the CR ≥ 70% group and the CR < 70% group (hazard ratio, 0.79; *p* = .100). Only the CR ≥ 90% group had a significantly lower risk of CCV death (hazard ratio, 0.56; *p* < .001) for those with complications. Medication adherence is significantly associated with CCV mortality during 10 years in newly diagnosed hypertensives patients. Patients with complications of hypertension have to continue a high adherence rate (CR ≥ 90) for better long‐term clinical outcomes.

## 1 INTRODUCTION

Hypertension is one of the most common chronic disease of the population aged over 30 years and is a major risk factor for cardio‐cerebrovascular (CCV) disease. In order to prevent complications of hypertension such as cerebral hemorrhage, cerebral infarction, heart failure and myocardial infarction, and CCV death, the use of antihypertensive medication has to be maintained indefinitely with life‐style modification. However, discontinuation of antihypertensive medication has been reported 50–60% after 6 months in randomized controlled studies^1^, ^2^ and compared with low adherent patients (proportion of days covered, ≤ 40%), high adherers (proportion of days covered, ≥ 80%) showed a decreased risk of cardiovascular event.^3^ It was also reported that patients with poor medication adherence (cumulative medication adherence, < 50%) had worse mortality from ischemic heart disease (IHD) (hazard ratio (HR), 1.64; 95% CI, 1.16–2.31), cerebral hemorrhage (HR, 2.19; 95% CI, 1.28–3.77), and cerebral infarction (HR, 1.92; 95% CI, 1.25–2.96) than those with good adherence (cumulative medication adherence, ≥ 80%).^4^ Adherence to antihypertensive medication is an indicator that can evaluate the degree of control of hypertension and has a characteristic that it varies depending on various factors such as the passage of time and the occurrence of complications. Nevertheless, early medication compliance after the initial diagnosis of hypertension is one of the main factors that can predict the risk of CCV death.

However, there has been little long‐term study to evaluate the impact of medication adherence on CCV mortality according to the presence and absence of hypertension complications. The purpose of this study is to evaluate the effect of medication adherence on CCV mortality in newly diagnosed hypertensive patients.

## 2 STUDY PATIENTS AND METHODS

### 2.1 Data source

This study analyzed sample cohort data of 1,108,369 people randomly extracted from the database of the Korean National Health Insurance Service (NHIS). Korean NHIS has nearly one hundred percent of the public's medical claims data between 2002 and 2016, including patient information (unique number, sex, year of birth, date of death, cause of death, disability, residential area, income, type of medical insurance, etc.) and claim materials (hospital visiting date, medical procedures, disease code, prescription code, etc.). The personal information of the individuals was not identified. This study was approved, and informed consent was waived by the Institutional Review Board of Yeungnam University Medical Center.

### 2.2 Study patients

**FIGURE 1:**
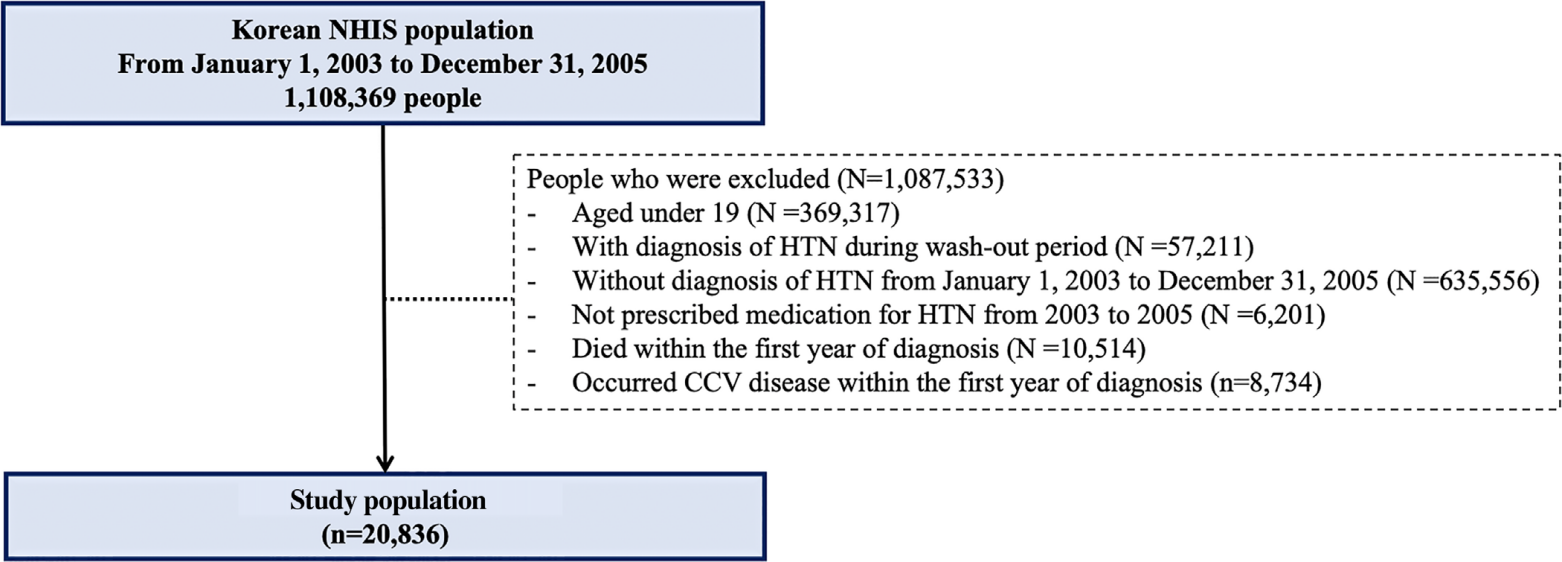
Flow diagram of the study population. NHIS, National Health Insurance Service; HTN, hypertension; CCV, cardio‐cerebrovascular

Among the 1,108,369 people for which Korean NHIS data existed in 2003 those under the age of 19 were excluded. Also, those diagnosed with hypertension during the wash‐out period (from January 1, 2002 to December 31, 2002) were excluded (*N* = 57,211). And only those who were newly diagnosed with hypertension from January 1, 2003 to December 31, 2005, were included. Among those diagnosed with hypertension between 2003 and 2005, those who had not been prescribed medication for hypertension were excluded (Figure 1).

In this study, when hypertension (*International Classification of Diseases, ICD*: I10) was marked as a major disease in the medical record, the patient was defined as hypertension. In addition, among patients who did not have a medical record of hypertension during the wash‐out period 2002, the first date on which antihypertensive drugs were prescribed between 2003 and 2005 was defined as the date of a new diagnosis of hypertension. For reference, antihypertensive drugs included 78 oral antihypertensive agents, including angiotensin‐converting enzyme inhibitor, angiotensin receptor blocker, diuretics, beta‐blockers, calcium channel blocker, and other antihypertensive agents (Table S1).

Then, among newly diagnosed patients with hypertension between 2003 and 2005, patients who died within 1 year after diagnosis of hypertension (*N* = 10,514) and patients with CCV disease within 1 year after diagnosis of hypertension (*N* = 8,734) were excluded (Figure 1). They were excluded for the purpose of assessing the long‐term effects of early antihypertensive medication adherence. For reference, CCV disease included hypertensive heart disease(ICD: I11), angina pectoris(ICD: I20), myocardial infarction(ICD: I21‐I23), chronic heart failure(ICD: I42, I50), cerebrovascular disease(ICD: I60‐I64) (Table S2).

In summary, the patients of this study were adult patients aged 19 years or older who were newly diagnosed with hypertension between 2003 and 2005 and started taking at least one hypertension drug at that time.

#### 2.2.1 Study design

The observation period of the study was defined as the period between an index date, the date of a new diagnosis of hypertension, and the end date of the observation. If a patient died during an observation period of up to 10 years, the date of death was considered the end of the observation. If the patient did not die, the observation period was considered 3,650 days.

Age, sex, type of medical insurance, medication adherence, presence of comorbidity, and family history were all calculated based on the index date. Through this, medication adherence over the first year was calculated based on the date each patient was diagnosed with hypertension in the actual practice, rather than on the period arbitrarily determined by the researcher.

**FIGURE 2:**
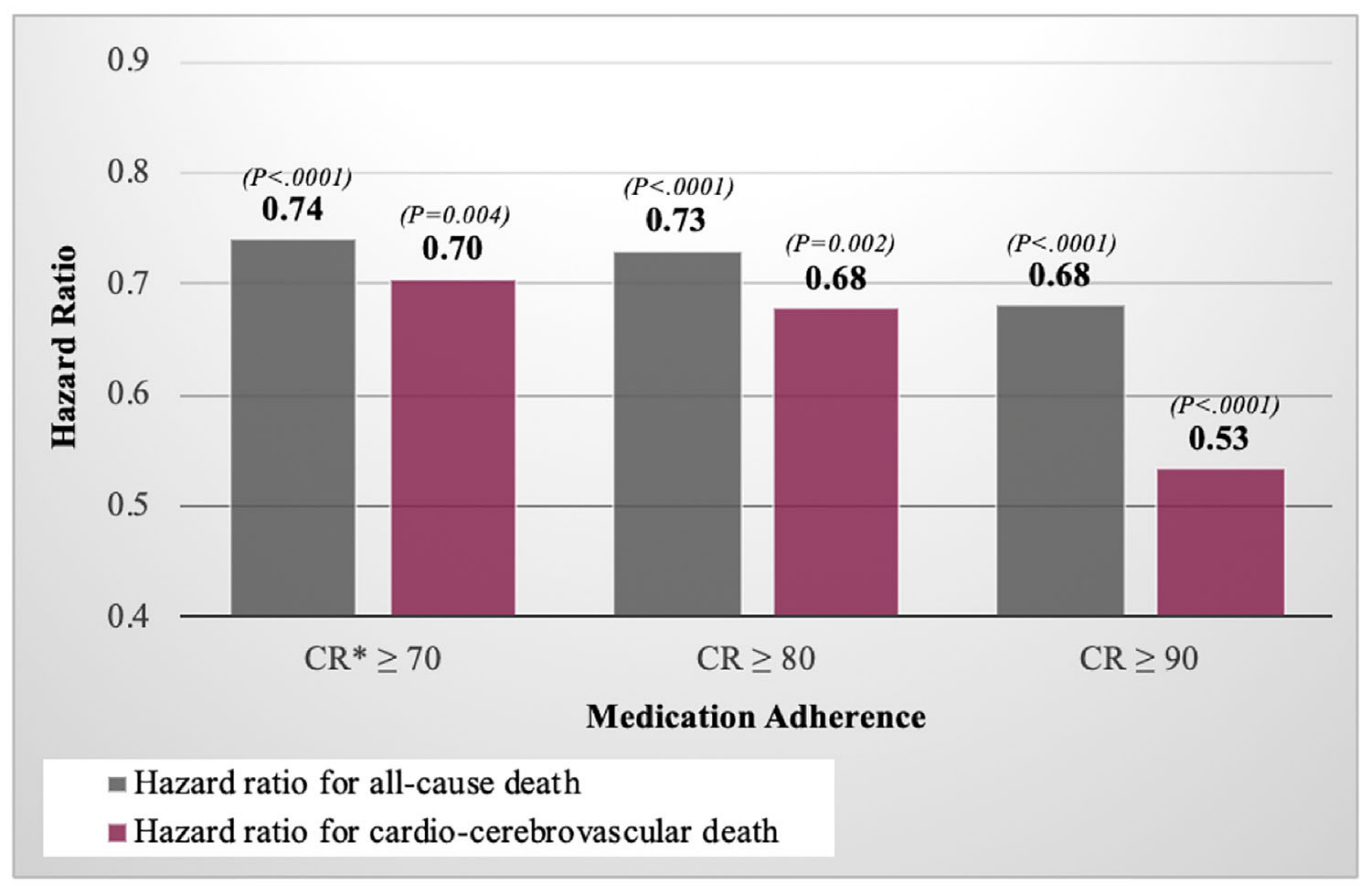
Association between medication adherence of antihypertensives and death. CR, compliance ratio; CCV, Cardio‐cerebrovascular

Study patients were divided into two groups according to their medication adherence to compare cerebrovascular mortality. We assessed how much decreased mortality in patients with adherence above a certain value compared to patients with adherence below a certain value. The compliance values were evaluated based on 70, 80, and 90, respectively (Figure 2).

**FIGURE 3:**
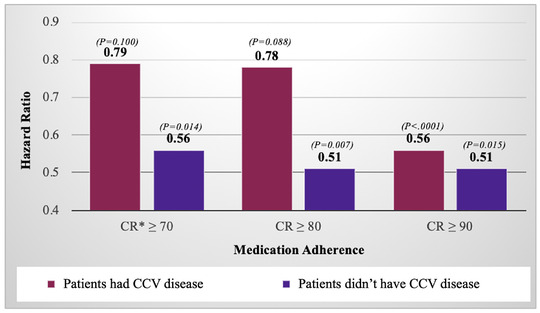
Association between medication adherence of antihypertensives and CCV death in patients who had or didn't have CCV disease. CR, compliance ratio; CCV, Cardio‐cerebrovascular

We also divided patients into two groups according to whether they had CCV disease. In our study, 9,792 patients had a CCV disease and 11,044 patients didn't have the CCV disease. Previously, the relative reduction in mortality due to high adherence was evaluated. In the second analysis, whether the patient had CCV disease was additionally considered (Figure 3).

### 2.3 Assessment of medication adherence

The proportion of days covered (PDC), is the recently preferred method of measuring medication adherence,^5^, ^6^ and we used compliance ratio (CR), which is a modified PDC for the purpose of our study. The PDC is calculated by the ratio of the number of days the patient is covered by the medication to the number of days in a uniform period. Using the PDC, drug adherence can be obtained by setting a uniform period (from January 1, 2003 to December 31, 2005) as a denominator. However, in this study, a date of a new diagnosis of hypertension is different for each patient. So we needed to modify the calculation formula to obtain drug adherence starting from the date of the new diagnosis of hypertension of each patient instead of using a fixed date. In addition, since the date of visit to the hospital is different for each patient, we used the earliest visit date 1 year later after the diagnosis to obtain drug adherence instead of using a uniform period. This does not mean that CR is a more accurate measuring method than PDC. We modified the PDC for our research purpose, and the formula we modified was the most similar to the previous CR calculation formula, so it was named as follows.^7^

We evaluated medication adherence by calculating CR using the date of the diagnosis of hypertension (day A), the earliest visit date 1 year later after the diagnosis (day B), and the number of days prescribed by hospitals and clinics.

It is calculated as follows:

$$\begin{array}{ccc} & & \mathrm{CR}\left(\mathrm{Compliance}\;\mathrm{Ratio}\right)\\ & & =\frac{Sum\;of\;the\;prescription\;period-\;a\;prescription\;period\;of\;day\;B\;}{The\;period\;between\;day\;B\;and\;day\;A}\times 100 \end{array}$$

During the period between day A and day B, if the patient visited the hospital or the clinic and was prescribed antihypertensive drugs, the sum of the prescription period was obtained by adding all the days of each prescription. We assumed that the patient visited the hospital after taking all the prescribed medications. Therefore, if the patient visited the hospital after a longer period than the prescribed number of days, it was assumed that the patient took all the drugs during the prescribed number of days and did not take them for the rest of the period. If the patient visited the hospital without completing the doctor's prescription days, it was assumed that the patient had taken all the medications by the day of the visit, and the medication adherence was not allowed to exceed 100%.

### 2.4 Definitions of CCV death

CCV death was defined according to the ICD code for the patient's causes of death. There are four major categories: cerebrovascular disease (I60–I69), IHD (I20–I25), chronic heart failure (I42, I50), and hypertensive disease (I10–I11). Cerebrovascular disease included subarachnoid hemorrhage (I60), intracerebral hemorrhage (I61), other nontraumatic intracranial hemorrhages (I62), cerebral infarction (I63), stroke (not specified as hemorrhage or infarction, I64), occlusion and stenosis of precerebral arteries (not resulting in cerebral infarction, I65), occlusion and stenosis of cerebral arteries (not resulting in cerebral infarction, I66), other cerebrovascular diseases (I67), cerebrovascular disorders in diseases classified elsewhere (I68) and sequela of cerebrovascular disease (I69). Ischemic heart disease included angina pectoris (I20), acute myocardial infarction (I21), subsequent myocardial infarction (I22), certain current complications following acute myocardial infarction (I23), other acute IHDs (I24), and chronic IHD (I25). Chronic heart failure included cardiomyopathy (I42) and heart failure (I50). Hypertensive disease included essential(primary) hypertension (I10) and hypertensive heart disease (I11) (Table S3).

### 2.5 Definitions of CCV disease

If CCV disease occurred during the observation period, the patient was considered to have had CCV disease. CCV disease was defined according to the ICD code in the medical record. For reference, CCV disease included hypertensive heart disease(ICD: I11), angina pectoris (ICD: I20), myocardial infarction (ICD: I21‐I23), chronic heart failure (ICD: I42, I50), cerebrovascular disease (ICD: I60‐I64)

### 2.6 Statistical analysis

The characteristics of patients who were clinically diagnosed with hypertension for the first time and who took antihypertensives for more than a year were described using frequency and percentage. We used statistical analysis such as T‐distribution, adjusted Cox proportional hazards regression model. A *p*‐value of less than .05 was chosen as a significant value in all statistical analyses.

In the Cox regression analysis, we adjusted for the age, sex, type of medical insurance, comorbidity (DM, dyslipidemia), family history (hypertension, strokes, heart disease), and whether CCV disease occurred during the observation period. We defined comorbidity as chronic diseases with high incidence or prevalence among those related to hypertension and included diabetes mellitus (ICD: E10–E14) and dyslipidemia (ICD: E78). Based on health examination data, the family history of hypertension, strokes, and heart disease was adjusted. All statistical analyses were performed with SAS version 9.4 (SAS Institute Inc., Cary, NC, USA).

## 3 RESULTS

### 3.1 Baseline characteristics

Baseline characteristics of 20,836 hypertensive patients according to 1 year of adherence to antihypertensive medication in this study were summarized in Table 1. 47.3% (*n* = 9,869) of the patients were male. In all patients, 10.3% (*n* = 2,139) died during an observation period up to 10 years, including 2.7% (*n* = 569) deaths from CCV disease. At least 1 year after diagnosis of hypertension, 54.9% (*n* = 11,440), 45.9% (*n* = 9,568), 32.8% (*n* = 6,835) of the hypertensive patients were adherent (CR ≥70%, ≥80%, and ≥90%). 47.0% (*n* = 9,795) of the hypertensive patients had CCV disease during the observation period while 53.0% (*n* = 11,041) of the patients didn't have CCV disease during that period. In the hypertensive patients with CCV disease, 404 (4.1%) patients died of CCV disease and in the patients without CCV disease, 165 (1.5%) patients died of CCV disease. Of the 9,795 patients with CCV disease, 23.3% (*n* = 4,848) of the patients had angina pectoris, followed by hypertensive heart disease (17.4%), heart failure (6.4%), and cerebrovascular disease (3.3%). Patients with diabetes mellitus or dyslipidemia were 26.4% (*n* = 5,511) and 31.9% (*n* = 6,643) when clinically diagnosed with hypertension. In all patients, 34.1% (*n* = 7,103) had history of hypertension, followed by strokes with 14.6% (*n* = 3,034) and heart disease with 6.5% (*n* = 1,349), but missing values were 21.8% (*n* = 4,547), 22.6% (*n* = 4,702) and 22.7% (*n* = 4,739) respectively.

**TABLE 1:** Baseline characteristics of study patients (*N* = 20,836)

	Without CCV disease(*N* = 11,041)		With CCV disease(*N* = 9,795)		
Characteristics	*N*	(%)	*N*	(%)	*p*‐value
Age (mean ± SD)	55.2 ± 11.8		58.6 ± 11.9		<.0001
Male	5,385	(48.8)	4,484	(45.8)	<.0001
Diabetes mellitus	2,643	(23.9)	2,868	(29.3)	<.0001
Dyslipidemia	3,423	(31.0)	3,220	(32.9)	.0038
Angina pectoris		–	4,848	(23.3)	
Cerebrovascular disease		–	683	(3.3)	
Heart failure		–	1,336	(6.4)	
Hypertensive heart disease		–	3,633	(17.4)	
Family history of heart disease	663	(6.0)	686	(7.0)	<.0001
Family history of hypertension	3,967	(35.9)	3,136	(32.0)	<.0001
Family history of strokes	1,626	(14.7)	1,408	(14.4)	.1013
Compliance groups					
CR ≥70	6,270	(56.8)	5,170	(52.8)	<.0001
CR ≥80	5,282	(47.8)	4,286	(43.8)	<.0001
CR ≥90	3,782	(34.3)	3,053	(31.2)	<.0001

The age, sex, and type of medical insurance are calculated based on index date (the date of clinical diagnosis of hypertension).

*Abbreviations*: CR, compliance ratio; CCV, Cardio‐cerebrovascular.

### 3.2 Clinical outcomes

The median follow‐up duration after the diagnosis of hypertension was 9.64 ± 1.26 years. The HR of CCV death was significantly lower in the CR ≥ 70% group than in the CR < 70% group (HR, 0.70; *p* = .004; Table 2). Similarly, the CR ≥ 80% and CR ≥90% groups had significantly lower HR for the cardiovascular mortality rate of 0.68 (*p* = .002; Table 2) and 0.53 (*p* < .0001; Table 2), respectively, than the CR < 80% and CR < 90% groups.

**TABLE 2:** Association between adherence to antihypertensive medication within 1 year after diagnosis and CCV death in all study patients

	Adherence within 1 year after diagnosis					
	CR ≥ 70		CR ≥ 80		CR ≥ 90	
Outcome (cause of death)	HR (95% CI)	*p*‐value	HR (95% CI)	*p*‐value	HR (95% CI)	*p*‐value
All‐cause death	0.74 (0.65‐0.83)	<.0001	0.73 (0.65‐0.83)	<.0001	0.68 (0.59‐0.78)	<.0001
CCV death	0.70 (0.56‐0.89)	.004	0.68 (0.53‐0.87)	.002	0.53 (0.40‐0.71)	<.0001
Cerebrovascular disease	0.69 (0.50‐0.95)	.022	0.69 (0.49‐0.96)	.029	0.56 (0.38‐0.84)	.004
Heart failure	0.89 (0.37‐2.15)	.789	0.64 (0.26‐1.62)	.348	0.11 (0.01‐0.79)	.028
Hypertensive disease	0.55 (0.26‐1.15)	.111	0.47 (0.21‐1.05)	.065	0.32 (0.11‐0.92)	.035
Ischemic heart disease	0.87 (0.55‐1.39)	.566	0.88 (0.55‐1.41)	.595	0.84 (0.50‐1.39)	.487

Each of HR of CR ≥70, CR ≥80, and CR ≥90 was calculated based on the mortality of the non‐compliance group CR < 70, CR < 80, and CR < 90 as a reference. HR was adjusted for the following covariates: age, sex, type of medical insurance, comorbidity (diabetes mellitus, dyslipidemia), family history (hypertension, strokes, heart disease), whether CCV diseases occurred during observation period.

*Abbreviations*: CR, compliance ratio; HR, hazard ratio; CCV, cardio‐cerebrovascular.

In the patients who didn't occur CCV disease during the observation period, the HR of CCV death was significantly lower in the CR ≥ 70% group than in the CR < 70% group (HR, 0.56; *p* = .014; Table 3). However, in patients with CCV disease during that period, there was no significant difference in risk of CCV death between the CR ≥ 70% group and the CR < 70% group (HR, 0.79; *p* = .100; Table 3). Of the patients with CCV disease, only the CR ≥ 90% group had a significantly lower risk of CCV death (HR, 0.56; *p* < .001; Table 3).

**TABLE 3:** Association between adherence to antihypertensive medication within 1 year after diagnosis and CCV death in patients who didn't have or had CCV disease

Hypertensive patients	Adherence within 1 year after diagnosis					
	CR ≥ 70		CR ≥ 80		CR ≥ 90	
	HR (95% CI)	*p*‐value	HR (95% CI)	*p*‐value	HR(95% CI)	*p*‐value
without CCV disease	0.56 (0.35‐0.89)	.014	0.51 (0.32‐0.83)	.007	0.51 (0.30‐0.88)	.015
with CCV disease	0.79 (0.60‐1.05)	.100	0.78 (0.58‐1.04)	.088	0.56 (0.39‐0.79)	<.0001

Each of HR of CR ≥70, CR ≥80, and CR ≥90 was calculated based on the mortality of the CR < 70, CR < 80, and CR < 90 group as a reference.

*Abbreviations*: CR, compliance ratio; HR, hazard ratio; CCV, cardio‐cerebrovascular.

## 4 DISCUSSION

This cohort study revealed that high adherence to antihypertensive medication can reduce the rate of all‐cause mortality and CCV mortality among newly diagnosed hypertensive patients. Furthermore, more intensive control of adherence to antihypertensive medication (CR ≥ 90) was associated with better clinical outcomes in all‐cause mortality and CCV mortality in patients with complications of hypertension. There was only a risk reduction in mortality from heart failure and hypertensive disease in CR ≥ 90 group and not in other groups. There was no statistical relationship between the HR of mortality from IHD and adherence to antihypertensive medications. In subgroup analysis, patients without complications of hypertension showed a similar degree of a risk reduction regardless of the degree of compliance if CR ≥ 70 was achieved. However, patients with complications showed a statistically significant risk reduction only in CR ≥ 90 group. Eventually, patients with complications of hypertension need more intensive control of adherence to antihypertensive medication (CR ≥ 90) for lowering CCV mortality than those presented without complications.

Present guidelines recommended an office blood pressure treatment target of < 140/90 mm Hg, irrespective of the level of cardiovascular risk, and comorbidities that patients have.^8^ In recent meta‐analysis, every 10 mm Hg reduction in systolic blood pressure significantly reduced the risk of major cardiovascular disease events (relative risk[RR] 0.80, 95% CI 0.77–0.83), coronary heart disease (RR 0.83, 95% CI 0.78–0.88), stroke (RR 0.73, 95% CI 0.68–0.77), and heart failure (RR 0.72, 95% CI 0.84–0.91).^9^ However, Benegas JR and colleagues and Chow CK and colleagues showed that less than 50% of patients achieved their target office systolic blood pressure of < 140 mm Hg.^10^, ^11^ In previous studies, there is growing evidence that poor adherence to antihypertensive medications has a relationship with poor blood pressure control and higher risk of cardiovascular events.^12^, ^13^, ^14^, ^15^, ^16^ On the contrary, Bramley TJ and colleagues showed high adherence (defined as medication possession ratio of 80%–100%) to antihypertensive medications was associated with a higher odds ratio (OR[95% CI], 1.45[1.04–2.02]) of blood pressure control compared with those with lower adherence.^17^ One study also reported that high adherence to antihypertensive medication is associated with a relevant decrease in cardiovascular events in the context of the primary prevention of cardiovascular diseases.^3^ In a recent meta‐analysis, it is confirmed that good adherence to antihypertensive medication was related to a lower risk of stroke (RR 0.73, 95% CI 0.67–0.79).^18^ In summing up the above studies, to achieve better clinical outcomes from CCV disease of hypertensive patients, it is of critical importance to keep better adherence to antihypertensive therapy. The results of our study are consistent with those of previous studies.

Most research has shown differences in clinical outcomes between patients of poor adherence to antihypertensive therapy and good adherence. However, our study showed that better adherence to antihypertensive medication has a greater relative risk reduction in reducing CCV mortality.

## 5 LIMITATIONS

This study has several potential limitations. Because this study is a retrospective cohort study, we showed that there is a relationship between high adherence to antihypertensive medication and a reduction in the rate of CCV mortality. However, it was unclear whether there is a causal relationship between them. The CR is one of the indirect methods to measure adherence to medication using an electronic pharmacy database.^19^ However, since there is no guarantee that the patients take all their prescribed medicine, actual adherence to antihypertensive medicine can be different from the CR. The recorded diagnosis using the *International Classification of Diseases (ICD)* from the database of the Korean NHIS is used for confirming the diagnosis of patients instead of medical records. This method can be different from the actual diagnosis. According to several studies, there is a possibility of ascertainment bias for the diagnosis of stroke due to the high fatality rate.^20^ And a diagnosis of acute myocardial infarction has a sensitivity ranging from 60% to 80%.^21^ We used the sample cohort extracted from the database of the Korean NHIS. There may be selection bias because there are a lot of missing data. In this study, patient factors such as psychological factors and level of knowledge of disease were not considered. Finally, chronic kidney disease is one of the major complications of hypertension and associated with a poor prognosis.^22^ However, from the data of this study, we couldn't confirm the information on this. Since the study population was newly diagnosed with hypertension, it seems that the number of patients with chronic kidney disease may account for a very small proportion of the total population.

## 6 CONCLUSIONS

In conclusion, high adherence to antihypertensive medication can reduce the rate of CCV mortality. Furthermore, patients presented with the presence of complications at initial diagnosis of hypertension need more intensive control of adherence to antihypertensive medication (CR ≥ 90) for lowering CCV mortality than those presented without complications.

## AUTHOR CONTRIBUTIONS

Conception and design: Cho‐Long Kim, Yoon‐Sung Do, Kyeong‐Soo Lee, Min‐Ah Nah, Tae‐Yoon Hwang. Cleaning of data: Yoon‐Sung Do, Cho‐Long Kim, Min‐Ah Nah. Data analysis: Cho‐Long Kim, Yoon‐Sung Do, Min‐Ah Nah. Data interpretation: Byung‐Jun Kim, Ung Kim, Jung‐Hee Lee. Manuscript writing: Cho‐Long Kim, Byung‐Jun Kim, Ung Kim, Jung‐Hee Lee, Kyeong‐Soo Lee. Final approval of manuscript: All authors.

## CONFLICT OF INTEREST

None.

## Supporting information

Supporting information

Supporting information

Supporting information
