# Supplementary figures and images for: Clinical impact of medication adherence on 10‐year cardio‐cerebrovascular mortality in newly diagnosed hypertensive patients
Source: J Clin Hypertens (Greenwich). 2021 Aug 12;23(9):1695–702. doi: 10.1111/jch.14320 (PMC8678795; doi:10.1111/jch.14320)

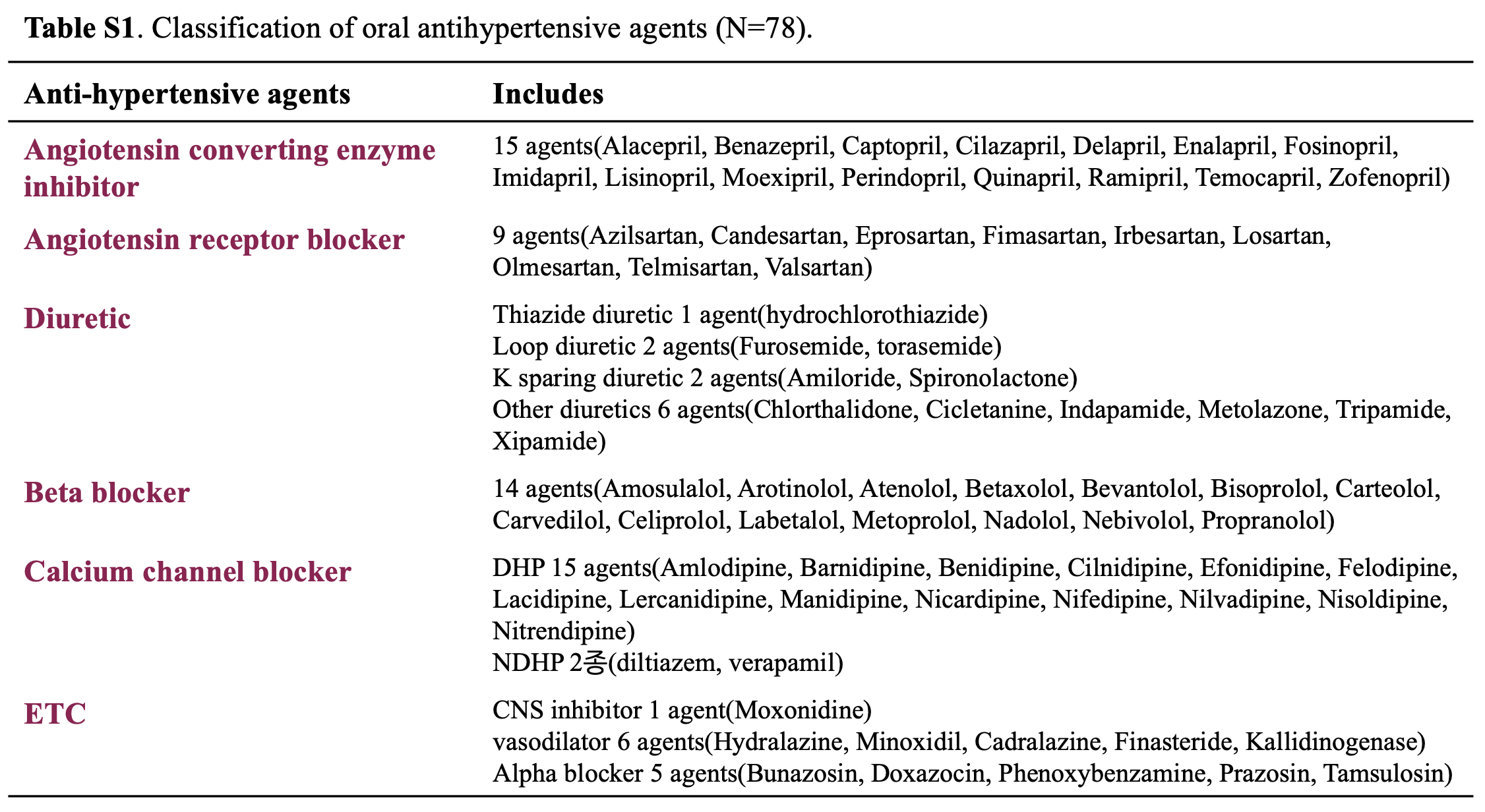

Supplement: Supplementary file 1 — Supporting information [file JCH-23-1695-s003.png]

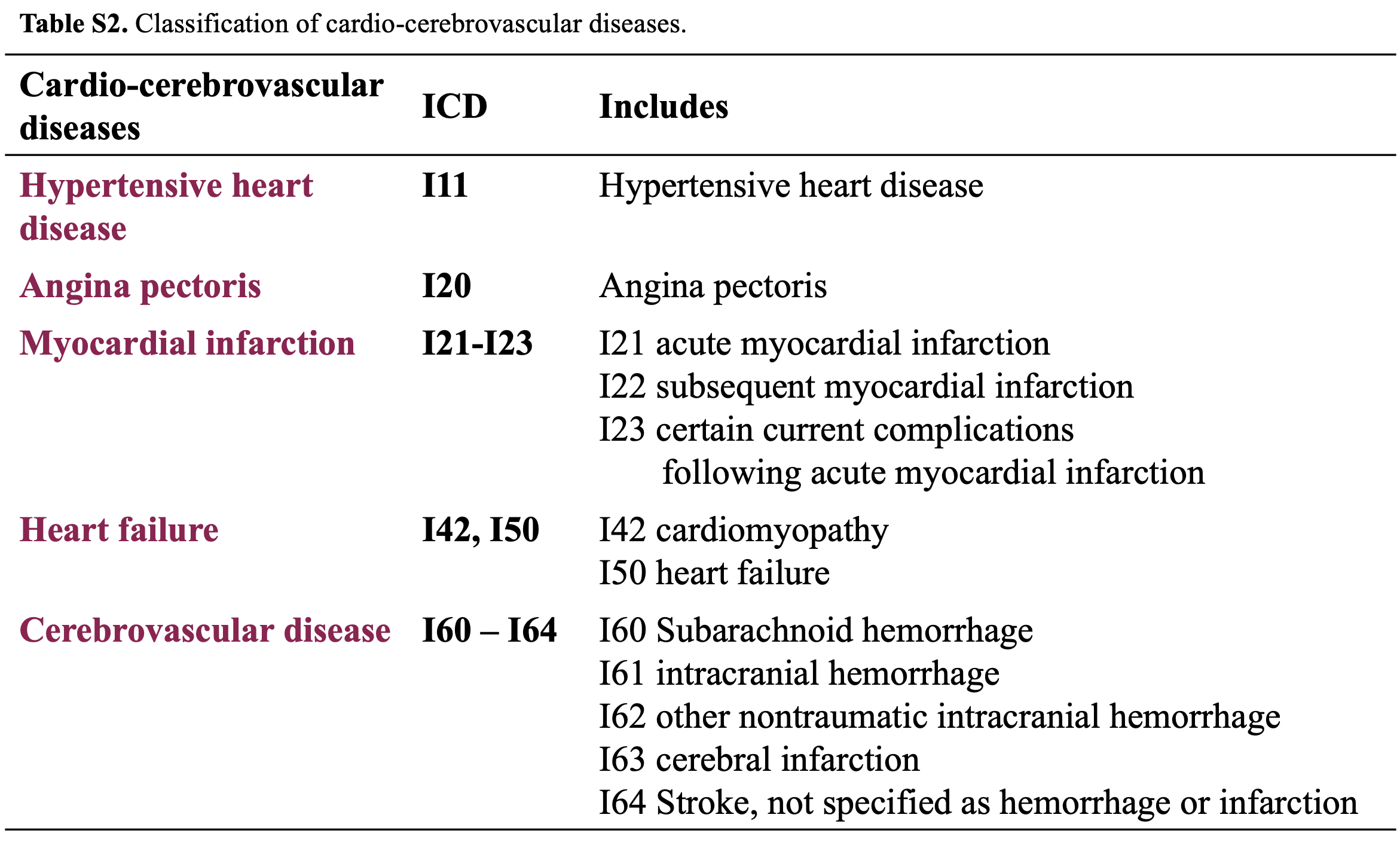

Supplement: Supplementary file 2 — Supporting information [file JCH-23-1695-s001.png]

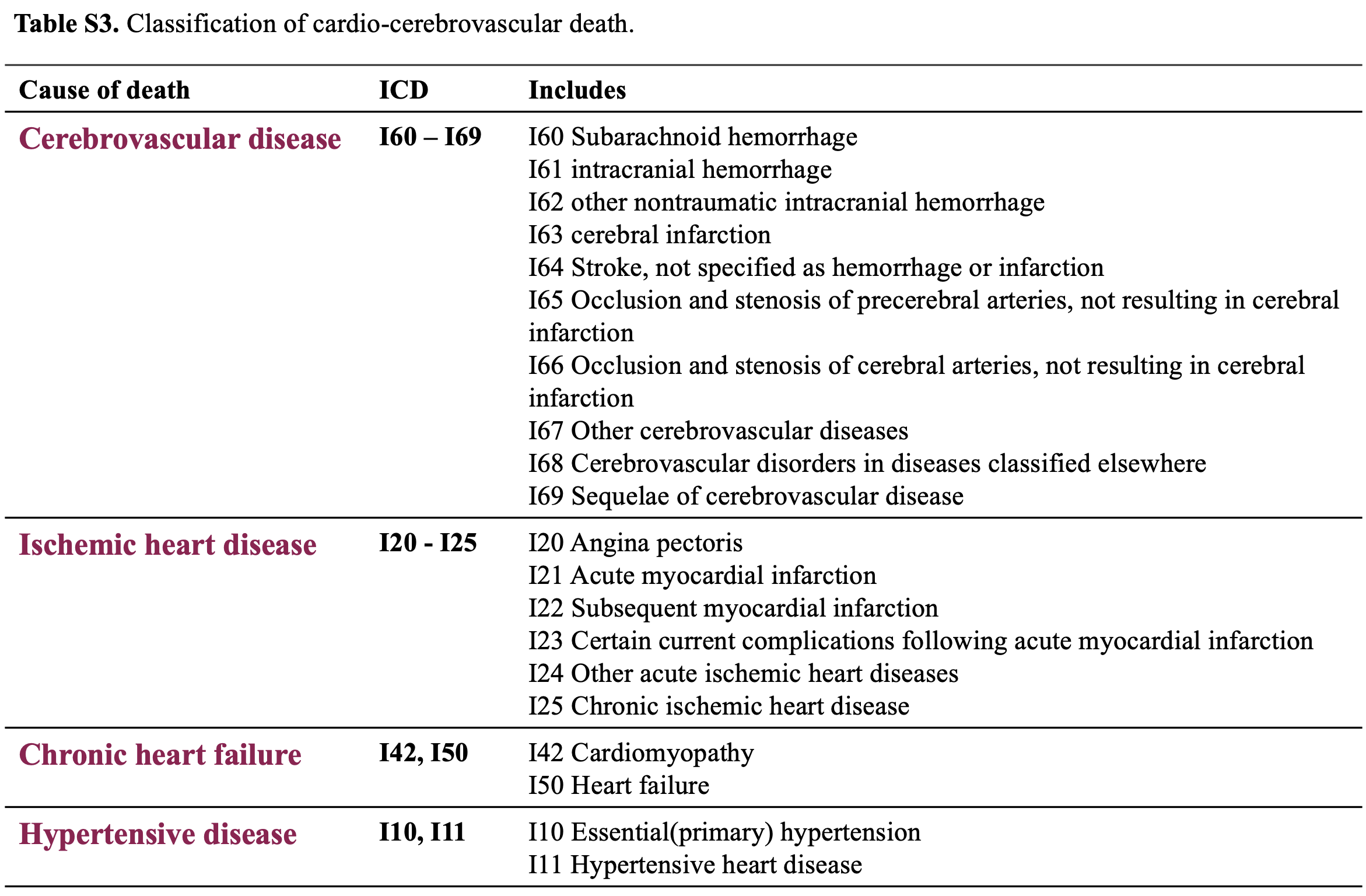

Supplement: Supplementary file 3 — Supporting information [file JCH-23-1695-s002.png]
